# Influence of the Structure of Perfluoroalkylsilanes Self-Assembled Monolayers on Tribological Properties of TiO_x_-Incorporated Diamond-like Carbon Coatings

**DOI:** 10.3390/molecules31173043

**Published:** 2026-08-30

**Authors:** Michał Cichomski, Barbara Burnat, Mariusz Dudek

**Affiliations:** 1 Department of Materials Technology and Chemistry, Faculty of Chemistry, University of Lodz, Pomorska 163, 90-236 Lodz, Poland; 2 Department of Inorganic and Analytical Chemistry, Faculty of Chemistry, University of Lodz, 12 Tamka St., 91-403 Lodz, Poland; barbara.burnat@chemia.uni.lodz.pl; 3 Institute of Materials Science and Engineering, Lodz University of Technology, Stefanowskiego 1/15, 90-924 Lodz, Poland; mariusz.dudek@p.lodz.pl

**Keywords:** TiO_x_-DLC coating, self-assembled monolayers (SAMs), perfluoroalkylsilane compounds, corrosion and tribological properties

## Abstract

This paper reports the effects of formed perfluoroalkylsilane self-assembled monolayers (SAMs) on the tribological and corrosion properties of TiO_x_-incorporated diamond-like carbon (TiO_x_-DLC) coatings deposited on a Ti6Al4V substrate. The SAMs were formed using 1H,1H,2H,2H -perfluorodecyltrichlorosilane (FDTS) and (3,3,3 -trifluoropropyl) trichlorosilane (FPTS) compounds. Their presence was confirmed using techniques such as ellipsometry, time-of-flight secondary ion mass spectrometry, and Fourier-transform infrared spectroscopy. The results of the ball-on-disc test indicate the role of the structure of the created SAMs on their tribological properties. The FDTS compounds with longer alkyl chains favor the creation of a well-packed layer bonded to the TiO_x_-DLC coating. This hydrophobic structure allows for obtaining the lowest coefficient of friction (0.180) during tribological tests. The results of electrochemical tests indicate that the SAM modification reduces the barrier properties of TiO_x_-DLC and provides enhanced kinetic stability against carbon matrix oxidation at higher anodic potentials.

## 1. Introduction

Contemporary engineering components are required to exhibit good mechanical, anti-corrosion, and tribological properties. Depending on the intended application and the bulk materials used, these surface demands can be met using thermochemical (carburizing, nitriding, and oxidizing) or mechanical (shot peening) processes and, ultimately, the deposition of an appropriate functional coating. Among the most interesting coatings are carbon-based (e.g., diamond-like carbon—DLC) coatings, which have applications in many fields, ranging from the automotive industry to biomedical engineering [1,2,3,4]. The properties of these coatings (mechanical, optical, electrical, and physicochemical) strongly depend on the deposition methods applied and the incorporation of other atoms (such as silicon and titanium) into their matrix [5,6,7,8,9,10,11,12]. Among physical and chemical vapor deposition methods, the latter, in the presence of plasma, is highly attractive. Plasma-enhanced chemical vapor deposition (PECVD) methods, especially DC pulsed PECVD and radio frequency (RF) PECVD techniques [13,14,15,16,17], allow for the modification of substrates with complex shapes (such as medical implants) using various carbon sources (e.g., methane, acetylene, benzene) and precursors, including titanium and silicon atoms (e.g., titanium (IV) isopropoxide, tetramethylsilane, hexamethyldisiloxane, silane). Such a variety of precursors allows for the arrangement of different process atmospheres, which, in connection with controlled negative self-bias (*V_B_*), makes it possible to synthesize coatings with the desired properties [16,17].

In prior work, we showed that, using the RF PECVD technique with methane and precursors such as hexamethyldisiloxane or tetramethylsilane, we can deposit Si-DLC coatings with better properties compared to pure DLC coatings deposited at the same total pressure and self-bias [17,18]. It was also shown that silicon atoms incorporated into the DLC coating act as adhesion centers between the carbon-based coating and the self-assembled monolayers (SAMs) formed on top of them, which further improves the tribological properties of the created layer system [19]. The role of SAMs as lubricant compounds was demonstrated for the TiO_x_-DLC coating deposited from a mixture of methane and a titanium (IV) isopropoxide precursor [20]. The obtained results showed that two types of compounds with different functional groups—n-decylphosphonic acid (DP) and 1H,1H,2H,2H-perfluorodecylphosphonic acid (PFDP)—on top of the TiO_x_-DLC coating significantly improved its tribological properties. The modified coating endured a 1000 m ball-on-disc test, while the unmodified coating wore out after 160 m under the same test conditions. This indicates that SAMs created by liquid deposition methods [21,22] can play an important role in contemporary engineering. A novelty of this work is that they allow the production of ultrathin fluoroalkylsilane layers, which influence the tribological properties and corrosion resistance of TiO_x_-DLC. Furthermore, this study attempted to combine RF PECVD technology to produce TiO_x_-DLC with vapor-phase deposition (VPD) technology, aiming to create a thin layer with controlled hydrophobicity. The novelty is the assessment of the effect of chain lengths on the chemical structure, hydrophobicity, corrosion, and tribological resistance of the coating produced using the VPD method.

In this work, titanium-oxide-incorporated carbon coatings deposited on a Ti6Al4V substrate by the RF PECVD method were modified with two perfluorosilane self-assembled compounds with different alkyl chain lengths: 1H,1H,2H,2H-perfluorodecyltrichlorosilane (FDTS) and (3,3,3-trifluoropropyl)trichlorosilane (FPTS). Fluorosilane SAMs are desirable for controlling surface hydrophobicity to limit or prevent undesirable protein adsorption or cellular interactions, which is crucial for the performance of many biomedical micro/nanodevices [23]. The modification was performed using the VPD method. The corrosion and tribological properties of the carbon-based coating before and after modification were investigated. The chemical structure of this layer system was characterized by time-of-flight secondary ion mass spectrometry (TOF-SIMS), Fourier-transform infrared spectroscopy (FTIR), and Raman spectroscopy.

## 2. Results and Discussion

The properties of TiO_x_-incorporated diamond-like carbon coatings deposited using the RF PECVD technique with methane and titanium (IV) isopropoxide mixture atmospheres were the subject of two publications [20,24]. It was shown that polarization curves of the 100 nm thick TiO_x_-DLC coating deposited on a Ti6Al4V substrate are strongly affected by the contents of incorporated titanium oxides in the DLC coating—the reactivity of the samples increases with an increasing amount of titanium and oxygen incorporated during the RF PECVD process into the deposited coating, and at the highest concentration, the formation of titanium oxide particles was observed [20]. Previously obtained results concerning the use of TiO_x_-DLC coatings as substrates for creating SAMs showed that the coatings contained 1.77 at. % of titanium and 2.19 at. % of oxygen, and they were characterized by 0.159 V of corrosion potential, 10.8 MΩ cm^2^ of polarization resistance, 14.5 GPa of hardness, a Young’s modulus of 145.7 GPa, and 0.74 GPa of tensile stress [20,24]. The TiO_x_-DLC coating was hydrophilic, with high contact angle hysteresis at 27.2° (advancing angle of 67.2° and receding contact angle of 40.0°).

**Figure 1:**
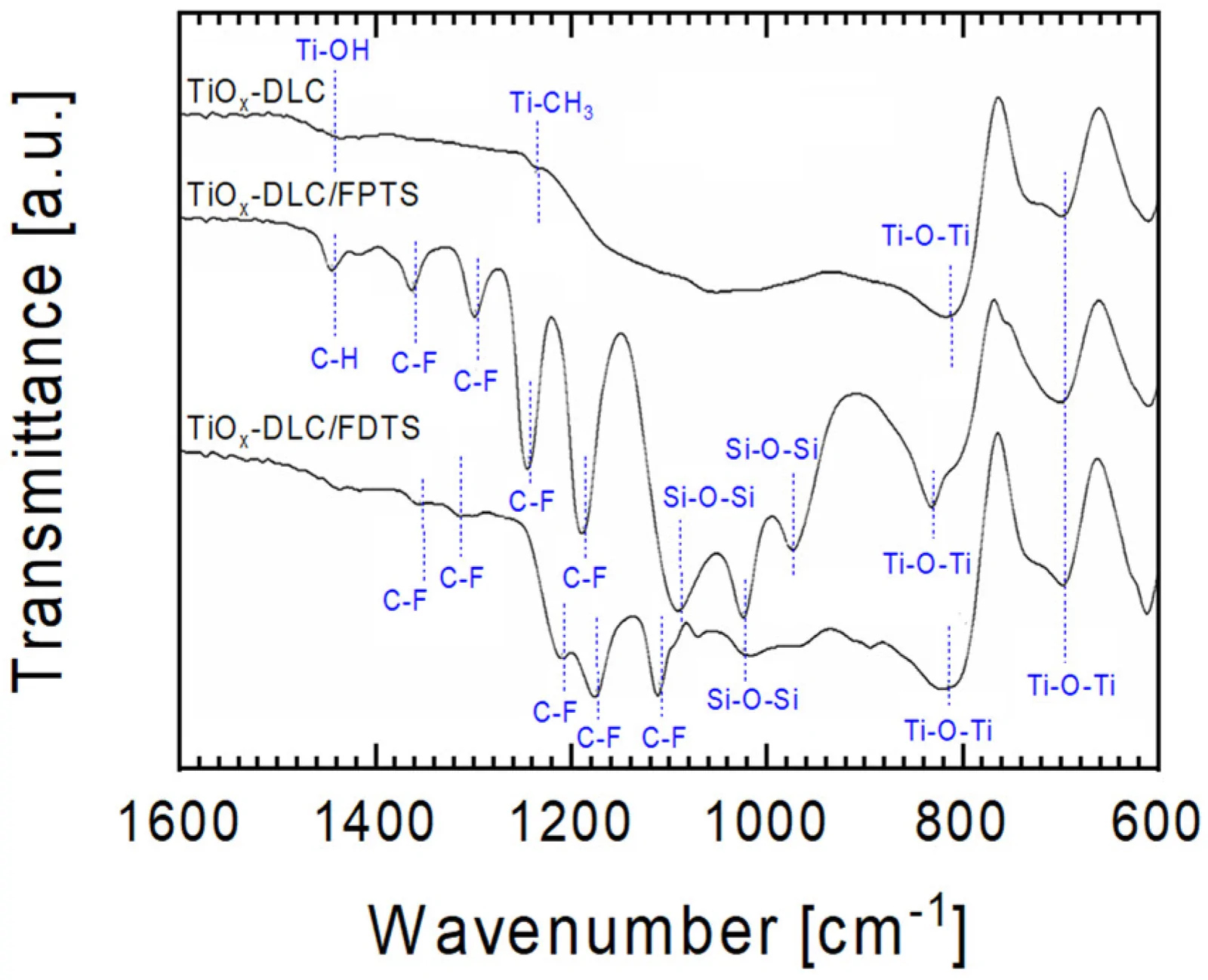
FTIR spectra of TiO_x_-DLC coatings before and after creation of SAMs of FPTS and FDTS compounds.

Confirmation of the creation of the TiO_x_-DLC coating self-assembled layers of FPTS and FDTS compounds was provided via FTIR spectra, as shown in Figure 1. Before modification, the coating spectra can be characterized by absorbing bands from Ti-OH bonds at about 1440 cm^−1^, Ti-CH_3_ bonds at about 1230 cm^−1^, and Ti-O-Ti bands at about 805 and 700 cm^−1^, confirming the presence of TiO_x_ in the structure of the DLC coating. After modification by perfluoroalkylsilanes, the recorded spectra (Figure 1) exhibit additional bands corresponding to the presence of Si-O-Si and C-F bonds. For FDTS-modified coatings, characteristic signals for Si-O-Si bond vibrations were present at about 1020 cm^−1^, and for FPTS, they were present at about 1090 cm^−1^, 1020 cm^−1^, and 965 cm^−1^. In the range of 1100 ÷ 1350 cm^−1^, for both perfluoroalkylsilane layers, stretching and bending of C-F bonds originating from fluoroalkyl chains are observed. Bands at 1440 cm^−1^ are due to C-H asymmetric stretching vibrations.

**Figure 2:**
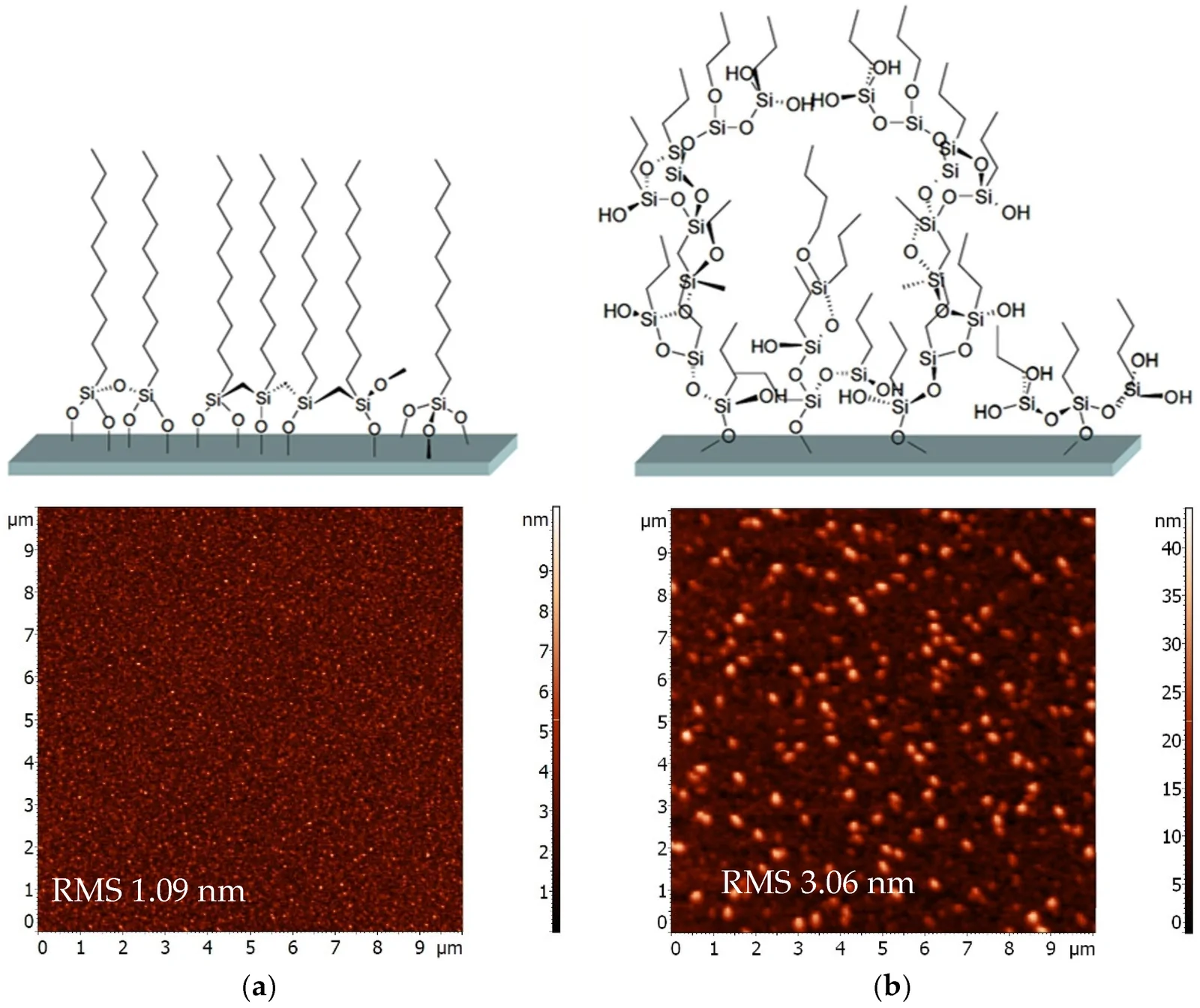
Structure of the forming SAMs with the example AFM map of their surfaces in the case of FDTS (**a**) and FPTS (**b**) components.

Secondary ion mass spectrometry with a time-of-flight ion analyzer was also used to confirm the creation of organosilicon SAMs (Table 1). Measurements performed after the modification process showed a reduction in the number of OH, Ti, and TiOH ions characteristic of the TiOx-DLC coating’s surface. This indicates that a significant portion of the surface hydroxyl groups was used for reactions with the silane headgroup (Si–Cl_3_). The large number of fragment ions containing fluorine atoms (CF_3_, CF_2_, CF, and F) visible after modification confirms the formation of organosilicon SAMs. Comparing the ion abundances of the FDTS and FPTS compounds containing three chlorine atoms in the headgroup but with different chain lengths, larger amounts of OH^−^ and SiOH^+^ fragment ions can be observed for the FPTS compound. These studies confirm the thesis that the volatile FPTS compound creates disordered structures and is capable of producing a coating several nanometers thick, and it consists of compound agglomerates containing a significant number of –SiOH groups not condensed with the substrate (Figure 2). In the case of FDTS, the lower abundances of OH^−^ and SiOH^+^ fragment ions than in the modification performed with FPTS indicate that the silanol groups formed in the hydrolysis reaction underwent condensation processes.

The proposed structure of the created SAMs is confirmed by maps of their surfaces (Figure 2) obtained by atomic force microscopy (AFM) and measurements of their thicknesses. The SAMs based on the FPTS compound exhibited a significantly greater thickness (5.09 nm) compared to the theoretical particle size (0.70 nm—determined using HyperChem 7.5 software [25]). This is due to the greater reactivity and volatility of this compound compared to the other organosilicon compounds tested, which leads to vertical polymerization. The increased reactivity is due to the headgroup structure containing three reactive chlorine atoms and the compound’s low molecular weight. This results in the hydrolysis of FPTS molecules, followed by the formation of siloxane bonds between adjacent molecules and the subsequent generation of agglomerates, which causes an increase in surface roughness (RMS 3.06 nm). The increase in thickness is a result of vertical polymerization and the formation of a highly disordered layer, which also causes higher contact angle hysteresis at 24.9° (92.5° advancing and 67.6° receding contact angles). A diagram of the FPTS SAMs, in which compound agglomerates are formed due to vertical polymerization and the formation of a disordered layer, is shown in Figure 2b. In the case of SAMs formed from FDTS molecules, which also contain three reactive atoms in the headgroup but are characterized by a longer perfluoroalkyl chain and a higher molecular weight, the thickness (3.11 nm) of the resulting coating is comparable to the theoretical size (1.64 nm) of the compound molecule. Therefore, siloxane bonds can only be formed in a horizontal plane (Figure 2a). This thesis is further confirmed by the lower contact angle hysteresis of 12.9° (with advancing and receding contact angles of 103.8° and 90.9°, respectively) and a surface roughness of 1.09 nm.

The corrosion resistance of the TiO_x_-DLC coatings, both before and after modification with perfluorosilanes (FPTS and FDTS), was evaluated based on the electrochemical parameters presented in Table 2.

The Ti6Al4V alloy coated with the unmodified TiO_x_-DLC coating exhibited the most noble behavior, characterized by the highest *E_cor_* (0.159 V) and a high *R_p_* (10.8 MΩ cm^2^). These values indicate a highly passive and compact barrier layer provided by the carbon-based matrix. Following the deposition of SAMs, a distinct transition in the electrochemical response was observed. Contrary to expectations, the incorporation of perfluorosilane SAMs led to a significant decrease in *E_cor_* and *R_p_* values compared to the pristine TiO_x_-DLC coating. Both modified samples showed a shift in *E_cor_* toward more cathodic (negative) values and a significant reduction in *R_p_*, which dropped to approximately 2.1–2.5 MΩ cm^2^.

This phenomenon can be attributed to the insufficient packing density of fluorinated chains, which fail to form a continuous diffusion barrier against Cl^−^ ions. Furthermore, the length of a fluorinated alkyl chain played a subtle but discernible role in the electrochemical response. The TiO_x_-DLC/FDTS sample, featuring a longer alkyl chain (10 carbons), exhibited a slightly higher *E_cor_* (−0.302 V) compared to the shorter-chained FPTS (−0.363 V). This can be explained by the stronger van der Waals interactions between the longer chains in FDTS, which promote the formation of a more ordered and densely packed monolayer, offering a marginally better shield against the electrolyte than the less organized FPTS layer.

However, the lower corrosion resistance of both SAM-modified samples may be associated with the VPD process itself. Considering that trichlorosilanes release HCl during hydrolysis and condensation, it can be hypothesized that this byproduct may influence the TiO_x_-DLC surface prior to complete SAM formation. Such an effect could contribute to the observed decrease in *R_p_*. However, no direct experimental evidence was obtained in the present study to unequivocally confirm this mechanism.

**Figure 3:**
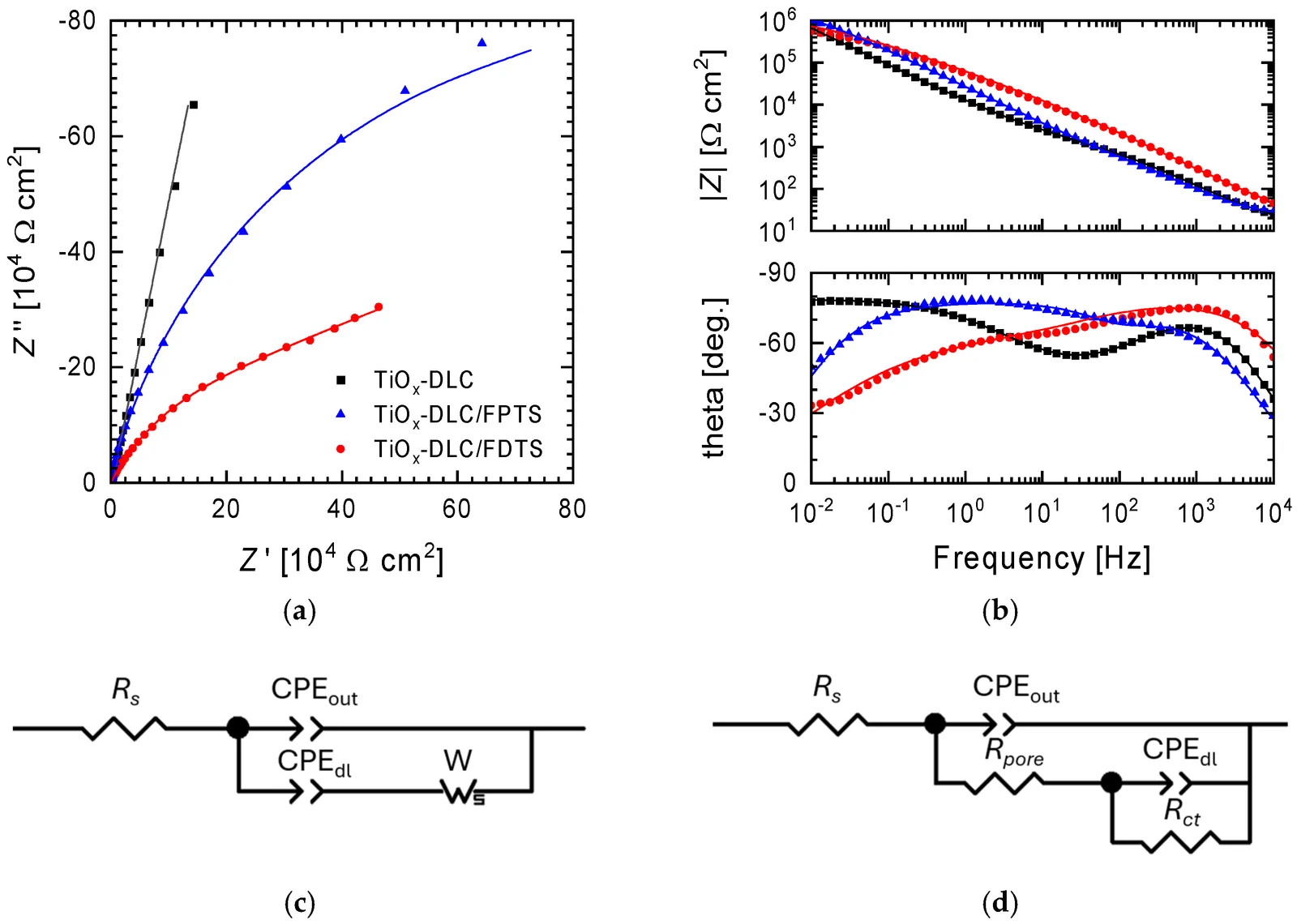
EIS spectra of TiO_x_-DLC coatings before and after modification with perfluorosilanes (FPTS and FDTS) recorded in 0.5 M NaCl solution: Nyquist (**a**) and Bode (**b**) plots (symbols—experimental data; solid lines—fitted spectra). Equivalent electrical circuits (EECs) used for fitting EIS data of unmodified TiO_x_-DLC (**c**) and SAM-modified TiO_x_-DLC (**d**) coatings.

The electrochemical structure and barrier properties of the coatings were further investigated using the EIS method. The obtained EIS data (Figure 3a,b) were fitted using equivalent electrical circuits (EECs) to quantify the dielectric and resistive components of the systems (Table 3), and the goodness of fit was verified by low chi-square (χ^2^) values. For pristine TiO_x_-DLC coatings, the best fit was achieved using a model with a Warburg element (Figure 3c), whereas the SAM-modified samples were fitted with a nested two-time-constant model (Figure 3d) to account for the additional silane barrier.

The pristine TiO_x_-DLC coating acts as a diffusion-limited barrier, as evidenced by the presence of the Warburg element (W). The diffusion resistance (*R_W_* = 4117 Ω cm^2^) and the relatively low time constant (*τ* = 0.34 s) suggest that the unmodified carbon matrix provides protection primarily by restricting the transport of corrosive ions through its narrow micropore network. In this state, the corrosion process is diffusion-controlled, which explains the high *R_p_* observed in previous tests. Upon modification with SAMs, a significant structural transition occurs. The disappearance of the Warburg element in both TiO_x_-DLC/FDTS and TiO_x_-DLC/FPTS samples indicates that the corrosion mechanism shifted from a diffusion-controlled to an activation-controlled process. The salinization process appears to have modified or “opened” the existing pore structure of the pristine coating, removing the diffusion limitation and making the substrate more directly accessible to the electrolyte. Consequently, the Warburg element is no longer physically applicable to the modified samples, and the nested two-time-constant model accurately reflects their activated state.

Comparisons between FDTS and FPTS reveal the influence of the alkyl chain’s length. The FDTS (longer chain) layer exhibited the lowest capacitance (*Q_out_* = 1.02·10^−6^ S·s^n^·cm^−2^), confirming the formation of a thicker or more dielectric barrier compared to FPTS. However, the *n_dl_* value for the double layer dropped significantly to 0.586. This very low exponent suggests a high degree of surface heterogeneity and supports the hypothesis of localized degradation caused by HCl during the VPD process, which significantly increased the roughness at the TiO_x_-DLC/electrolyte interface. In contrast, the FPTS-modified sample showed a much higher capacitance (*Q_out_* = 6.45·10^−6^ S·s^n^·cm^−2^) and lower pore resistance (*R_pore_* = 2594 Ω cm^2^). These values indicate that the shorter fluorinated chains fail to form a dense, protective monolayer, leaving the DLC surface more exposed. Interestingly, its *n_dl_* remained high (0.937), suggesting less interfacial damage than in the case of the longer-chained FDTS.

**Figure 4:**
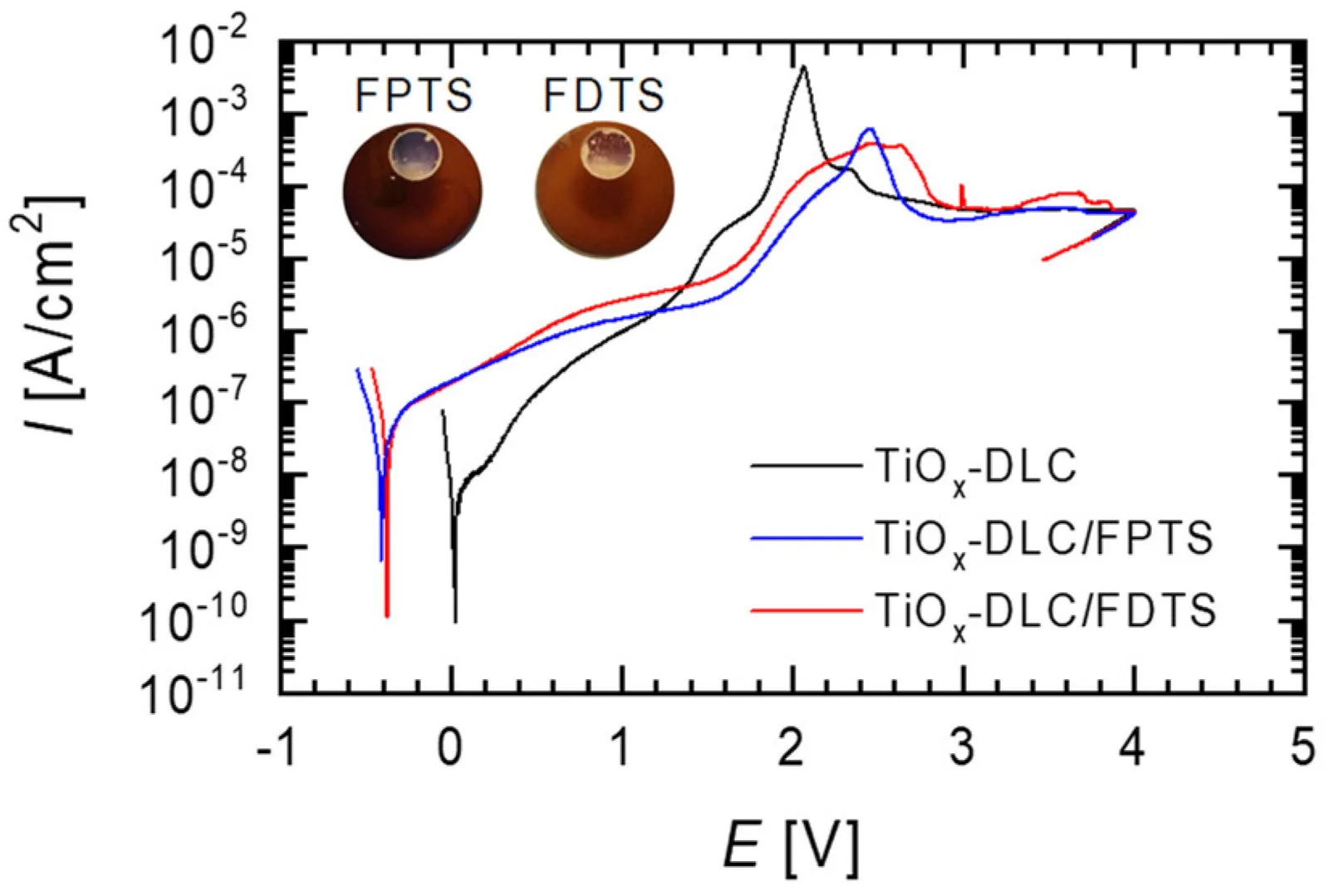
The polarization curves of Ti6Al4V coated with TiO_x_-DLC coatings modified by FPTS and FDTS in 0.5 M NaCl (scan rate 1 mV/s). Within the figure are photos of samples with FPTS and FDTS SAMs after corrosion tests.

The increased electrochemical reactivity of the modified samples was also evident under anodic polarization (Figure 4). The applied perfluorosilanes influenced the course of the polarization curve, particularly above 1.5 V, where anodic peaks associated with the electrochemical oxidation of the carbon layer were observed. For the unmodified TiO_x_-DLC sample, these peaks appeared in the potential range of 1.0–2.5 V. Interestingly, after modification with FPTS and FDTS compounds, these peaks were shifted to higher potentials and showed reduced intensity. This suggests that the presence of SAMs may kinetically hinder the oxidation process of the carbon matrix at higher potentials. Post-corrosion microscopic observations revealed no evidence of corrosion damage on the substrate. Instead, a partial removal of the modified coatings was observed, leaving a characteristic blue interference coloration on the surface, indicative of the formation of a stable passive oxide layer on the Ti6Al4V substrate, which effectively protected the metal from corrosion even after partial degradation of the carbonaceous layer. According to the literature, anodic oxide films formed on the Ti6Al4V alloy exhibit a highly complex chemical nature. They often possess a stratified structure consisting of inner and outer layers that contain not only titanium oxides but also aluminum and vanadium oxides [26,27]. However, the precise composition and distribution of these species strongly depend on the specific electro-oxidation conditions and the local electrolyte environment.

**Figure 5:**
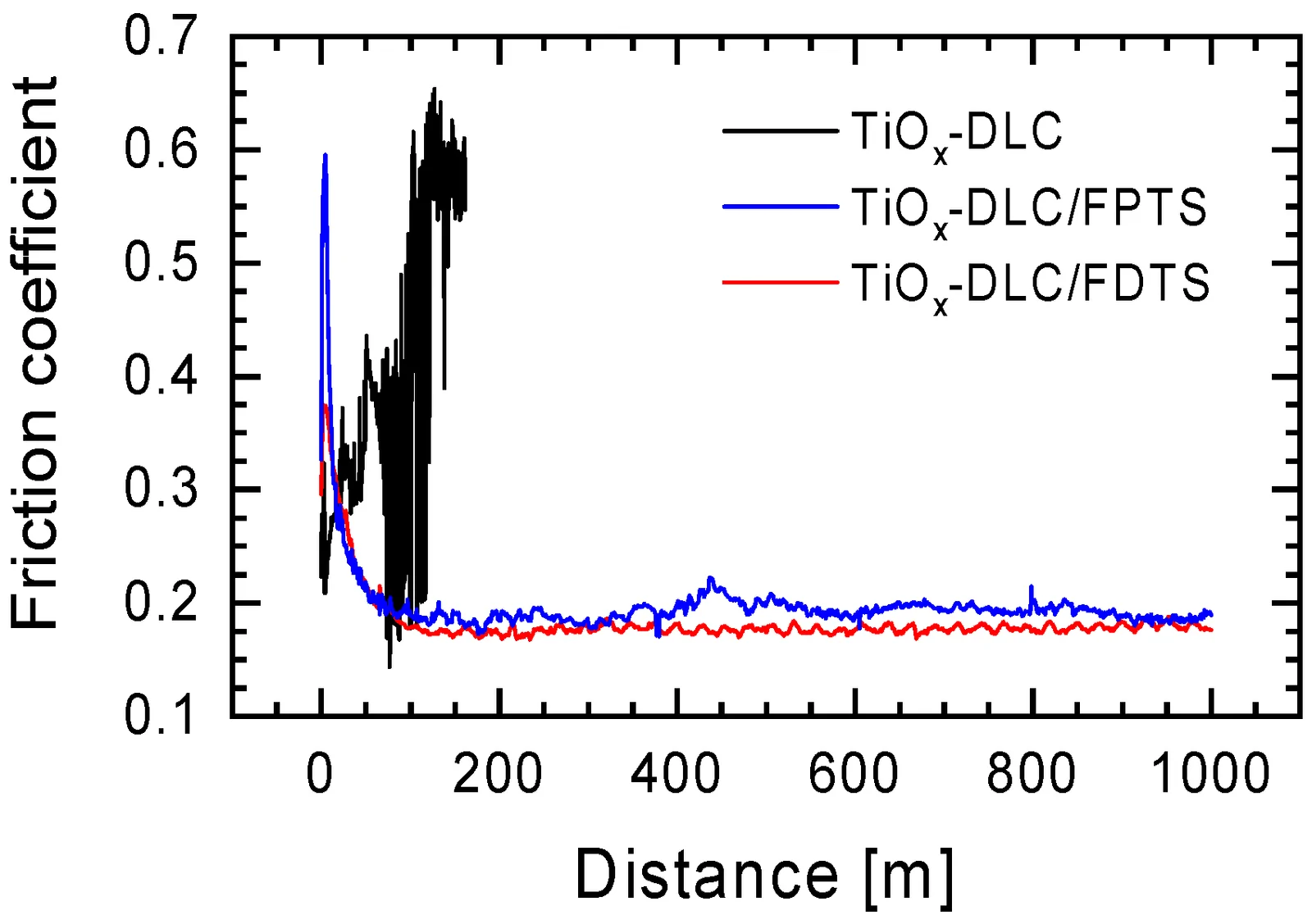
Evolution of the friction coefficient of pure TiO_x_-DLC coatings in comparison with coatings modified by FPTS and FDTS compounds during ball-on-disc tests with a Si_3_N_4_ ¼-inch ball.

**Figure 6:**
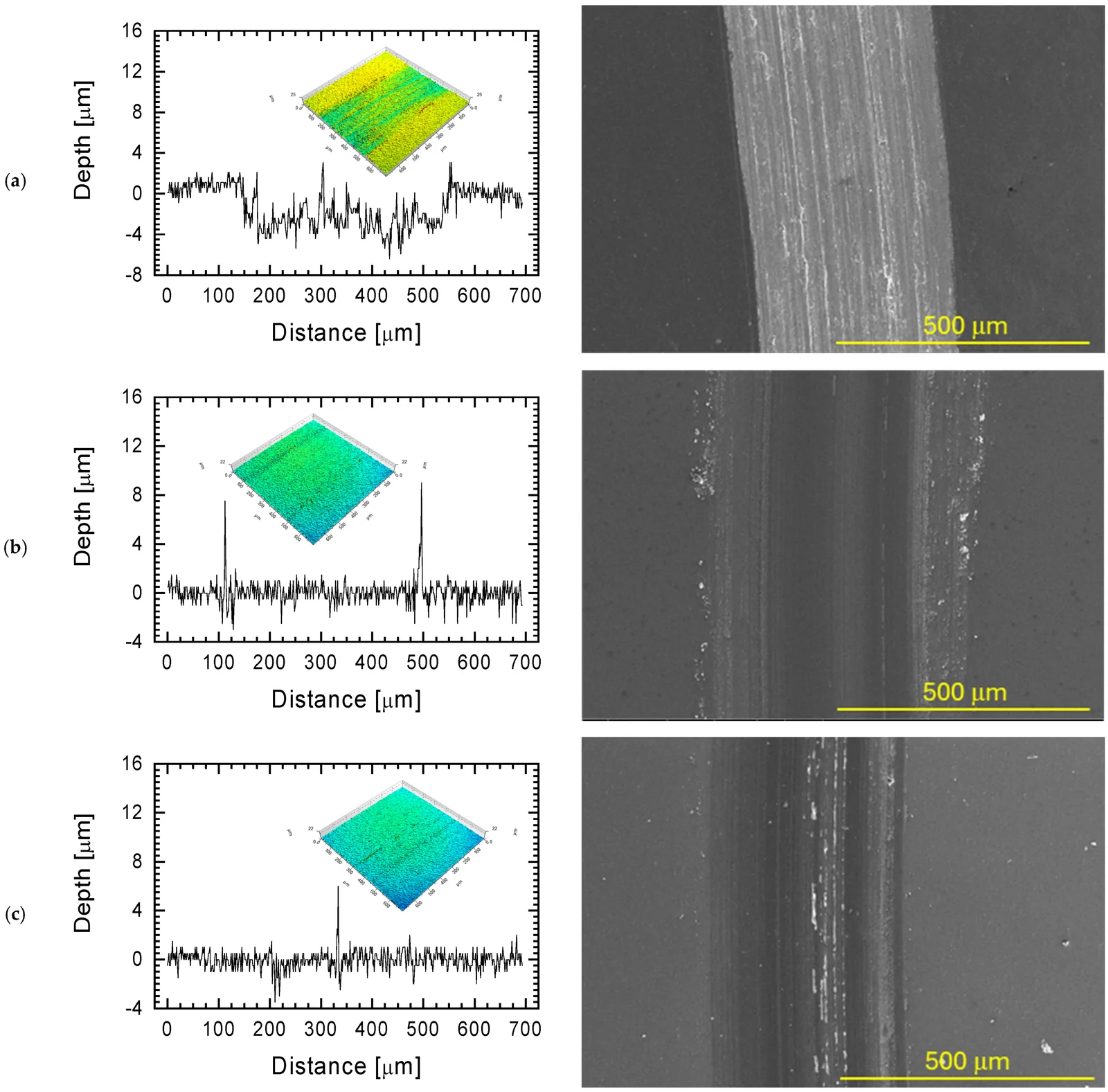
Profile (obtained on the basis of a 3D image from a confocal microscope) and SEM image of the wear trace of TiO_x_-DLC coatings without modification (**a**) and after modification by FPTS (**b**) and FDTS (**c**) components.

The tribological properties of TiO_x_-DLC coatings without and with created FPTS and FDTS compound layers were examined using a ball-on-disc test with a ¼-inch-diameter Si_3_N_4_ ball as the counterbody. The investigation shows that the unmodified TiO_x_-DLC coating possesses poor tribological properties [20]. The ball-on-disc test at 160 m distance was interrupted because the measured friction coefficient achieved a value typical for the friction of a Si_3_N_4_ ball on titanium alloy (Figure 5). Visual assessments of the wear trace showed the Ti6AL4V alloy substrate. The measured depth of the wear trace reached a value of 4 μm, which is 4 times higher than the thickness of the deposited TiO_x_-DLC coating. Figure 6 shows the profile of the wear trace obtained on the basis of a 3D image from CLSM measurements. The TiO_x_-DLC surface after modification presented a smooth profile with small amounts of debris formed at the boundary of the wear trace, which was clearly visible in the case of the TiO_x_-DLC/FPTS layer system (Figure 6b).

The poor tribological properties of the TiO_x_-DLC coating did not prevent the achievement of low friction coefficients after the formation of DP and PFDP layers (0.176 and 0.173, respectively) on the TiO_x_-DLC coating [20]. Figure 5 shows that, in the case of the TiO_x_-DLC coating with FPTS and FDTS layers created on top, it survived the ball-on-disc test with average friction coefficients equal to 0.186 and 0.180, respectively, after the initial period of testing. These indicate that the TiO_x_-DLC/FDTS layer system presents better tribological properties than the FPTS modifications due to having the lowest coefficient of friction and most stable evolution of the friction coefficient over time (Figure 5). Naked-eye examinations of the wear trace allow the observation of a small, rarely distributed or discovered area of the Ti6Al4V substrate. This observation is confirmed in images from CLSM and SEM measurements (Figure 6)—in particular, EDS analysis confirms the existence of a small area of the Ti6Al4V substrate in the wear trace of the modified TiO_x_-DLC coating. The concentration of the discovered area of the Ti6Al4V substrate is higher in the case of the TiO_x_-DLC/FDTS layer system. To explain the behavior of modified perfluorodecyltrichlorosilane compounds, it is worth returning to the previously discussed structure of SAMs (Figure 2) and the water contact angle of the tested surfaces. The structure of FPTS SAMs (5.09 nm thick) was described as the vertical polymerization of FDTS compounds and the formation of a highly disordered layer. The structure of FDTS SAMs (3.11 nm thick) was described as a horizontally oriented FDTS component. FDTS SAMs were hydrophilic (103.8° advancing and 90.9° receding contact angles), while FPTS SAMs exhibited this characteristic only at the initiation of the contact-angle hysteresis measurement (92.5° advancing and 67.6° receding contact angles), and the surface of the TiO_x_-DLC coating was always hydrophilic (67.2° advancing and 27.2° receding contact angles). The hydrophilic nature of the TiO_x_-DLC coating’s surface with high contact-angle hysteresis (40.0°) resulted in a high coefficient of friction (Table 4) and rapid surface wear during the ball-on-disc test (Figure 6a). The use of perfluorodecyltrichlorosilane compounds, regardless of the SAM structure, significantly increased the contact angle while simultaneously reducing its hysteresis (originally hydrophobic surface), leading to low wear (Figure 6b,c) and a low friction coefficient (Table 4) compared to the hydrophilic surface of the TiO_x_-DLC coating. The lowest wear and friction coefficient values were obtained for the hydrophobic TiO_x_-DLC/FDTS structure with horizontally oriented SAMs. This result is consistent with the results of Yang et al. [28].

**Figure 7:**
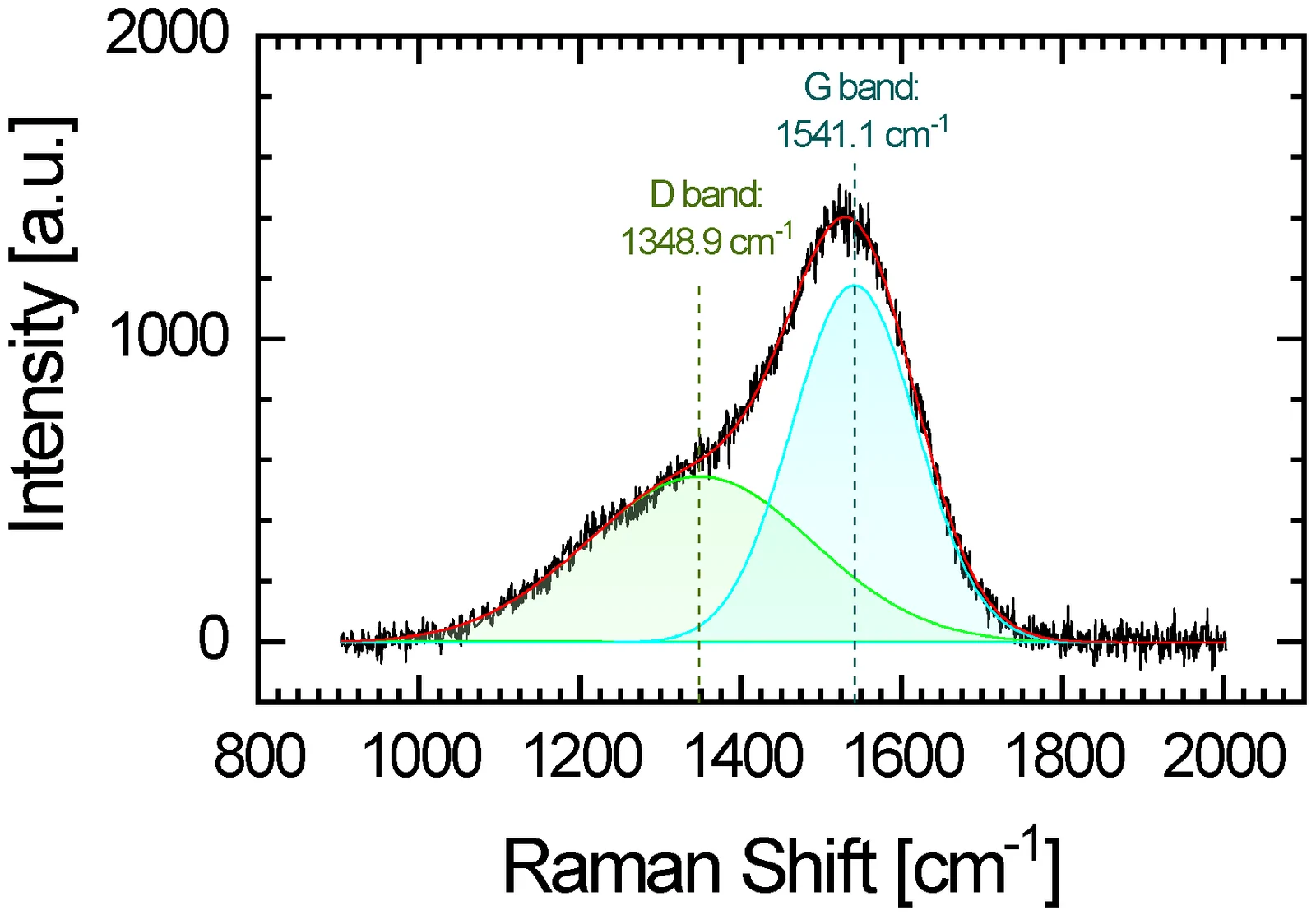
Example of deconvolution of the Raman spectra of TiO_x_-DLC coatings modified by FDTS components. The spectrum was collected from the wear trace after the ball-on-disc test.

Raman spectroscopy measurements of the TiO_x_-DLC coating with created SAMs at the wear trace and areas not affected by the tribological test showed that the obtained spectra, which exhibited two main features, were almost identical. In Figure 7, an example of a spectrum for the wear trace of the TiO_x_-DLC/FDTS system after deconvolution into two bands is shown (the broad G peak and D peak—characteristic of amorphous carbon structures rich in *sp*^2^ bonds). The detailed analyses of the results of the spectrum deconvolution—G band position and determined *I_D_*/*I_G_* ratio—allow for the identification of subtle changes in the investigated samples. Table 4 shows the values of this ratio and the G band position determined for the spectra collected at the center of the wear trace and at a sample area not affected by the tribological test, compiled with the average value of the friction coefficient determined for the last 100 m of the ball-on-disc test.

The value of the *I_D_*/*I_G_* ratio indicates the degree of modification of the TiO_x_-DLC coating by FDTS and FPTS compounds because, in both cases, this ratio was lower for the SAM-modified TiO_x_-DLC coatings than for the unmodified coating (Table 4). At the same time, the G band position shifts to a lower frequency value. In the case of the wear trace, the *I_D_*/*I_G_* ratio decreased from 0.68 to 0.54 after the ball-on-disc test for TiO_x_-DLC/FPTS structures, whereas for the TiO_x_-DLC/FDTS structure, it increased from 0.74 to 0.84. The same tendency is observed in the case of the G band position: For the TiO_x_-DLC/FPTS structure, a shift to a lower frequency of ~3 cm^−1^ was observed, whereas for TiO_x_-DLC/FDTS, a shift of about 3 cm^−1^ to a higher frequency was observed. This effect is related to the graphitization process of the coating as a result of the temperature increase during the friction test—the well-ordered, horizontal FDTS layer led to better thermal conductivity and finally to the graphitization process. These processes led to a lower and more stable friction coefficient over time (Figure 5).

## 3. Materials and Methods

### 3.1. TiO_x_-DLC Coating Deposition Process

Titanium-oxide-incorporated diamond-like carbon (TiO_x_-DLC) coatings modified by fluoroalkylsilane compounds were deposited on a Ti6Al4V substrate (one-inch diameter and 6 mm thick disc) via a radio frequency plasma-enhanced chemical vapor deposition (RF PECVD) process previously described in [20,24]. The sample surfaces prior to the RF PECVD process were ground on sandpaper with gradations between 80 and 2000, and they were polished with colloidal silica suspension using an automatic polishing machine; the surface roughness (*R_a_*) was equal to 0.021 ± 0.001 μm (measured by a Hommel Tester T1000 profilometer HOMMEL ETAMIC, Villingen-Schwenningen, Germany). Based on previously described properties of the TiO_x_-DLC coating [20,24], the deposition was performed under 400 V negative self-bias and 20 Pa pressure of a mixture of methane (CH_4_) and titanium (IV) isopropoxide (Ti[OCH(CH_3_)_2_]_4_) atmospheres. Methane flow was controlled by a mass flowmeter, whereas Ti[OCH(CH_3_)_2_]_4_ was controlled by a needle valve. After establishing a flow of methane, a Ti-containing precursor was introduced to determine the total operating pressure at a value of 20 Pa. The thickness of the coating dedicated for corrosion investigation was approximately 100 nm, whereas for the tribological test, it was about 1000 nm (in order to improve adhesion to the substrate, prior to deposition of TiO_x_-DLC coatings dedicated for the tribological test, a Ti interface layer with a thickness of 100 nm was deposited using the magnetron sputtering technique with respect to a Ti target). Before the deposition process, samples were cleaned and activated by Ar ion sputtering in a high-intensity RF plasma discharge for 10 min at a negative self-bias of 1100 V and residual pressure of 4 Pa. The TiO_x_-DLC coating contained 1.77 at.% of titanium and 2.19 at.% of oxygen.

### 3.2. SAMs Formation Process

The self-assembled monolayers were formed by two perfluoroalkylsilane compounds: 3,3,3-trifluoropropyl trichlorosilane (FPTS—97%, ABCR, GmbH and Co. KG, Karlsruhe, Germany) and 1H,1H,2H, 2H-perfluorodecyltrichlorosilane (FDTS—97%, ABCR, GmbH and Co. KG Karlsruhe) were deposited on the surface of the TiO_x_-DLC coating using the vapor phase deposition (VPD) method. Prior to the formation of SAMs, in order to remove organic contaminants, the TiO_x_-DLC coating was subjected to low-pressure air plasma (Diener Electronic Plasma-Surface-Technology, GmbH & Co. KG. Ebhausen, Germany, Zepto, 40 Hz, 100 W). The plasma environment also enables the formation of hydroxyl and Ti-O groups, playing the role of anchoring centers for modifying compounds necessary for the creation of stable covalent bonds between the modifier molecules and coating surface [29,30]. The VPD processes were performed at low pressure (ca. 1 × 10^−3^ Pa) at controlled laboratory temperatures. TiO_x_-DLC coatings with adsorbed FPTS and FDTS compounds were purged in argon and finally heated at 60 °C for 24 h under normal pressure. Details concerning the optimization of FPTS and FDTS deposition procedures, including parameters such as time, temperature, and pressure, as well as reactions of fluoroalkylsilanes, are reported in our previous publication [25].

### 3.3. Characterization Methods

The chemical structure of the SAMs formed on the TiO_x_-DLC substrate was investigated using a Nicolet iS50 FTIR spectrophotometer equipped (Thermo Scientific, Madison, WI, USA) with an ultra-high-sensitivity, low-noise, linearized mercury–cadmium–telluride (MCT) detector. A VariGATR™ grazing angle ATR accessory (Harrick Scientific Products, Inc., Pleasantville, NY, USA) with a Ge ATR crystal, specifically designed for the analysis of thin layers (including monolayers on semiconductor and/or metallic substrates), was applied at an incidence angle of 65° ± 1°. All spectra were recorded in the spectral range of 600–4000 cm^−1^ with a resolution of 8 cm^−1^ by accumulating 32 scans under a continuous flow of dry air; a background spectrum of pure silicon (32 scans) was recorded prior to the measurements. Additionally, the chemical structure of the SAMs was studied using a ToF-SIMS IV mass spectrometer (Ion-ToF GmbH, Münster, Germany). During the measurements, the analyzed area was irradiated with pulses of Bi^3+^ primary ions with an energy of 25 keV, a repetition rate of 10 kHz, and an average ion current of 0.14 pA. The analysis time was 100 s for both positive and negative secondary ions, ensuring an ion dose that was well below the static limit of 10^13^ ions/cm^2^. The analyzed surface area was 100 µm × 100 µm.

The prepared layer structures were also investigated by Raman spectroscopy. The tests were performed using a Renishaw inVia Raman Microscope equipped (Renishaw, Wotton-under-Edge, UK) with a 532 nm laser arranged in a backscattering geometry. The investigated wavenumber ranged from 750 to 2100 cm^−1^. All measurements were carried out in air at room temperature.

The thicknesses of the SAMs were determined using spectroscopic ellipsometry (J. A. Woollam V-VASE ellipsometer, J.A. Woollam Co., Lincoln, CA, USA). Measurements were conducted at a 70° angle of incidence over a wavelength range of 260–1300 nm. A Cauchy dispersion model was used to calculate the thickness of the SAMs. The surface morphology of SAMs was characterized using atomic force microscopy (AFM-Solver P47 system, NT-MDT, Moscow, Russia) in tapping mode under ambient conditions. The scan area was fixed at 10 μm × 10 μm. The root-mean-square (RMS) roughness parameter was calculated from AFM images. To ensure repeatability, the measurements were performed three times.

The water contact angle of the TiO_x_-DLC substrate with SAMs was also evaluated. The contact angle hysteresis was measured at room temperature using a drop shape analyzer (DSA25, Krüss GmbH, Hamburg, Germany). Drops with a volume of 1.5 to 3 µL were dispensed automatically using a microsyringe. The advancing and receding contact angles were determined automatically using the instrument’s software (ADVANCE, Krüss GmbH, Hamburg, Germany). To ensure repeatability, the measurements were performed three times.

Electrochemical corrosion measurements were carried out using a PGSTAT 30 potentiostat with an FRA2 module (EcoChemie, Autolab, Utrecht, The Netherlands) in a deoxygenated 0.5 M NaCl solution maintained at 37 ± 1 °C. The standard three-electrode measuring setup consisted of the following: (*i*) the sample with an exposed area of 0.64 cm^2^ as the working electrode; (*ii*) a saturated calomel electrode (SCE, *E* = 0.236 V_SHE_) as the reference electrode; and (*iii*) a platinum gauze as the counter electrode. The corrosion potential (*E_cor_*) was determined by monitoring the open-circuit potential for 2000 s until steady-state conditions were achieved. The value of polarization resistance (*R_p_*) was determined according to the Stern–Geary method in a narrow potential range (±20 mV versus *E_cor_*) with a scan rate of 0.166 mV/s. *R_p_* was calculated as the inverse of the slope of the current density vs. potential plot near the open-circuit potential using CorrView software version 3.5e (Scribner Associates Inc., Southern Pines, NC, USA). Electrochemical Impedance Spectroscopy (EIS) was carried out in a frequency range from 10 kHz to 0.01 Hz using an AC signal amplitude of 10 mV. The impedance spectra, consisting of 50 points, were recorded at *E_cor_*. The obtained data were subsequently analyzed using ZView v.2.90 software (Scribner Associates, Inc.). Furthermore, potentiodynamic polarization curves were recorded over a broad anodic range (up to +4 V) at a scan rate of 1.0 mV/s to characterize the anodic behavior of the samples. To ensure the reliability and reproducibility of the results, all electrochemical measurements were performed in triplicate.

Tribological properties of investigated coatings were characterized using a tribometer (CSM) working in ball-on-disc configuration. A Si_3_N_4_ ball having a diameter of ¼in was used as the counterbody. The sliding speed was set to 0.1 m/s over a distance of 1000 m under a load of 1 N, with a sliding radius of 10 mm. All tests were performed under dry friction conditions at a temperature of 23 °C and relative air humidity of 30%. For repeatability purposes, the measurements were performed three times.

The surface morphology of the wear tracks was visualized using a Nova NanoSEM 450 field emission scanning electron microscope (FEI, Hillsboro, WA, USA) equipped with a Schottky gun. The elemental analysis of wear tracks was performed using energy-dispersive spectroscopy (EDS). The data were collected using an EDAX X-ray spectrometer (AMETEK B. V.; Tilburg, The Netherlands) equipped with an Octane Pro Silicon Drift Detector (SDD) at accelerating voltages of 5 kV and 20 kV. The wear tracks were also examined using a Nikon confocal laser scanning microscope (CLSM), (Nikon Corporation, Tokyo, Japan).

## 4. Conclusions

TiO_x_-DLC coatings, containing 1.77 at.% titanium and 2.19 at.% oxygen, were deposited on a Ti6Al4V substrate using the RF PECVD method. Subsequently, self-assembled monolayers (SAMs) of FDTS and FPTS components were formed on top of the coatings. The successful deposition and modification processes were confirmed by FTIR, ToF-SIMS, water contact angles, corrosion, and tribological measurements. Ball-on-disc tests against a Si_3_N_4_ ball (counterbody) demonstrated that the structures modified with FPTS and FDTS (hydrophobic surfaces) survived the full 1000 m sliding distance, exhibiting average friction coefficients of 0.186 and 0.180, respectively, after the initial run-in period. In contrast, the test for the unmodified TiO_x_-DLC coating (hydrophilic surface) was interrupted at 160 m due to coating failure. The structure of the formed SAMs plays a crucial role in determining the properties of the prepared samples. The FDTS compounds, which possess longer alkyl chains, preferentially form horizontally oriented structures relative to the investigated surface. Conversely, the shorter FPTS components favor vertical orientation, resulting in a multi-layer SAM structure. The better-packed, horizontal structure of the FDTS layer results in strong bonding to the TiO_x_-DLC substrate and yields a hydrophobic surface, which provides the lowest friction coefficient during tribological testing. Finally, electrochemical results indicate that the SAM modification reduces the barrier properties of the TiO_x_-DLC system in chloride media. However, the presence of SAMs was found to kinetically hinder the oxidation of the carbon matrix at higher anodic potentials.

## Figures and Tables

**Table 1:** Results of measurements obtained using secondary ion mass spectrometry with a time-of-flight ion analyzer for organosilicon SAMs created on the surface of TiO_x_-DLC coatings. All values presented in the table represent numbers of counts divided by 10^3^.

Fragmented Ions	Number of Positive Ions			Number of Negative Ions		
	TiO_x_-DLC Coating	FPTS Modification	FDTS Modification	TiO_x_-DLC Coating	FPTS Modification	FDTS Modification
CF_3_	-	2.5	700.8	-	-	-
CF_2_	-	-	-	-	132	721
CF	-	-	-	-	2.4	756.5
F	-	-	-	-	163.2	1482.3
OH	-	-	-	121.2	17.7	277.6
TiOH	90.2	46.1	11.1	-	-	-
Ti	150.8	31.3	16.1	-	-	-
SiO_2_	-	-	-	-	100.8	27.6
SiOH	-	81.6	6.2	-	-	-

**Table 2:** Corrosion parameters of TiO_x_-DLC coatings deposited on Ti6Al4V substrates before and after modification by FPTS and FDTS compounds.

Sample	Corrosion Potential, *E_cor_* [V]	Polarization Resistance, *R_p_* [MΩ cm^2^]
TiO_x_-DLC	0.159 ± 0.009	10.8 ± 1.4
TiO_x_-DLC/FPTS	−0.363 ± 0.007	2.5 ± 0.5
TiO_x_-DLC/FDTS	−0.302 ± 0.025	2.1 ± 0.3

**Table 3:** Electrochemical parameters obtained by fitting the EIS spectra with equivalent electrical circuits for the pristine and SAM-modified TiO_x_-DLC coatings. The presented parameters correspond to the fitting of a representative curve for each sample group. All values are normalized to the geometric surface area.

Sample	*R_s_*	CPE_out_		*R_pore_*	CPE_dl_		W			*R_ct_*	Χ^2^
		*Q_out_*	*n_out_*		*Q_dl_*	*n_dl_*	*R_W_*	*τ*	*α*		
TiO_x_-DLC	17.9	2.45 × 10^−6^	0.922	-	1.45 × 10^−5^	0.867	4117	0.340	0.241	-	2.01 × 10^−5^
TiO_x_-DLC/FDTS	21.1	1.02 × 10^−6^	0.923	4219	4.51 × 10^−6^	0.586	-	-	-	1.03 × 10^6^	9.95 × 10^−4^
TiO_x_-DLC/FPTS	21.8	6.45 × 10^−6^	0.847	2594	1.16 × 10^−6^	0.937	-	-	-	1.95 × 10^6^	1.64 × 10^−3^

*R_s_*—Solution resistance [Ω cm^2^]; CPE_out_—outer layer constant phase element with *Q_out_* value [S·s^n^·cm^−2^] and *n_out_* exponent; *R_pore_*—pore resistance of the SAM [Ω cm^2^]; CPE_dl_—interfacial (double layer) capacitance with *Q_dl_* value [S·s^n^·cm^−2^] and *n_dl_* exponent; W—Warburg impedance; *R_W_*—Warburg resistance [Ω cm^2^]; *τ*—Warburg time constant [s]; *α*—Warburg exponent; *R_ct_*—charge transfer resistance [Ω cm^2^].

**Table 4:** Results of friction coefficient estimation for the last 100 m of the test and deconvolution of Raman spectra obtained from sample areas at the center of the wear trace and free of wear damage—G band position and *I*_D_/*I*_G_ ratio.

Sample	Friction Coefficient	No Damaged Surface		Center at Wear Trace	
		G Band Position [cm^−1^]	*I*_D_/*I*_G_	G Band Position [cm^−1^]	*I*_D_/*I*_G_
TiO_x_-DLC	0.581 ± 0.036 ^a^	1539.8 ± 0.4	0.97 ± 0.04	-	-
TiO_x_-DLC/FPTS	0.186 ± 0.003	1538.3 ± 0.8	0.68 ± 0.03	1535.2 ± 1.2	0.54 ± 0.02
TiO_x_-DLC/FDTS	0.180 ± 0.003	1537.5 ± 0.8	0.74 ± 0.04	1541.1 ± 0.7	0.84 ± 0.05

^a^ The last 40 m of the test.

## Data Availability

The original contributions presented in this study are included in the article. Further inquiries can be directed to the corresponding author.
